# Practical and cost-effective model to build and sustain a cardio-oncology program

**DOI:** 10.1186/s40959-020-00063-x

**Published:** 2020-07-16

**Authors:** Diego Sadler, Chakra Chaulagain, Beatrice Alvarado, Robert Cubeddu, Elizabeth Stone, Thomas Samuel, Bruno Bastos, David Grossman, Chieh-Lin Fu, Evan Alley, Arun Nagarajan, Timmy Nguyen, Wesam Ahmed, Leah Elson, Zeina Nahleh

**Affiliations:** 1 Cleveland Clinic Florida, Heart and Vascular Center, 2950 Cleveland Clinic Blvd, Weston, FL 33331 USA; 2 Cleveland Clinic Florida, Maroone Cancer Center, 2950 Cleveland Clinic Blvd, Weston, FL 33331 USA

**Keywords:** Cardio-oncology, Cardiology, Oncology, Screening, Care delivery model

## Abstract

**Background:**

Cardio-Oncology (CO) is a new subspecialty that thrives mostly in large academic quaternary centers. This study describes how to establish a successful cardio-oncology program, with limited resources, in order to effectively manage the unique care required by this patient population.

**Methods:**

Clinical data was collected from 25 consecutive months. There were four foundational elements to establish a CO program: 1. Clinical program: integrating staff and resources from the Heart and Vascular, and Cancer Centers; 2. Education Program: establishing a platform to educate/advocate with respect to CO; 3. Engagement with professional societies: active engagement allowed for the successful establishment of the proposed CO program; and 4. Research program: establishing data collection modalities/cooperation with other institutions.

**Results:**

474 consecutive patients were treated by our CO program during the first 25 months of operation. Clinical data, information about cancer treatment, cardiovascular co morbidities, cardiac testing and impact of CO management are reported.

**Conclusions:**

A successful CO program can be established utilizing existing resources without the need for significant additional assets. Integration with professional societies, advocacy, education and research, provide a platform for learning and growth. This model improves access to care and can be reproduced in a variety of settings.

## Background

There are currently 16 million cancer survivors in the United States [1], and one-quarter of them may die from cardiovascular disease (CVD) [2]. Cancer patients have a 2–6 times higher CVD mortality risk than the general population, and CVD mortality is evident throughout the continuum of cancer care with an acute early risk phase and a chronic phase [3]. The importance of the cardiovascular care for these patients is increasingly recognized. Furthermore, for those patients with access to effective cancer treatments and declining cancer mortality, CVD management becomes critical to improve outcomes and reduce overall mortality [3, 4]. It is in this subset of patients where cardio oncology may have its greatest impact.

There are multiple factors that may lead to decreased healthcare access and poor clinical outcomes for many of these patients, including: the lack of knowledge regarding the association between cancer and heart disease, lack of early detection of potentially cardio-toxic effects of certain cancer-related treatments, the prevalence of an aging population amongst cancer survivors [5].

Cardio-oncology (CO) is a rapidly growing subspecialty in the United States and throughout the world [6]. However, despite its momentum, CO programs exist predominately in large, academic, quaternary institutions [7]. The reason for this limited setting is a result of multiple challenges related to institutional resources. The American College of Cardiology (ACC)‘s National Cardio-Oncology Survey [6], identified specific barriers which might limit the implementation of CO programs, including: lack of funding, limited interest, lack of infrastructure, and lack of educational opportunities [6]. Additional sources have identified that limited available mentoring in CO has also been a negative contributing factor [8].

Additionally, only nine cardiovascular (CV) fellowship programs, nationwide, reported offering fully structured formal training in CO [9]. This lack of access to training may directly contribute to the observed, limited proportion of cardiologists specifically involved in this specialty [10].

CV testing indications that are not traditionally covered by medical insurance may also present a barrier to establishing a successful CO program. Examples of non-reimbursed services include: post-radiation non-invasive cardiac testing for surveillance, biomarkers during chemotherapy treatment [11], strain imaging, and cardiac magnetic resonance (CMR) used for early detection of cardiac effects of cancer-related therapies. These, and other non-reimbursed services, may result in an increased financial burden associated with the implementation and management of CO programs.

As the disease burden of oncologic therapy-induced CV complications increases, there is a corresponding need for established CO programs. Therefore, we present a practical model that allowed us to rapidly establish a CO program at our institution. We also describe the patient characteristics and needs assessed in this population, for the first 25 months of formal clinic operation and data collection. Finally, we describe how the utilization of existing resources contributed to a successful build-out and sustainability of our program without the need of any significant investment, as overseen by both the Heart and Vascular Center and the Cancer Center.

## Methods

### The program components

**Fig. 1:**
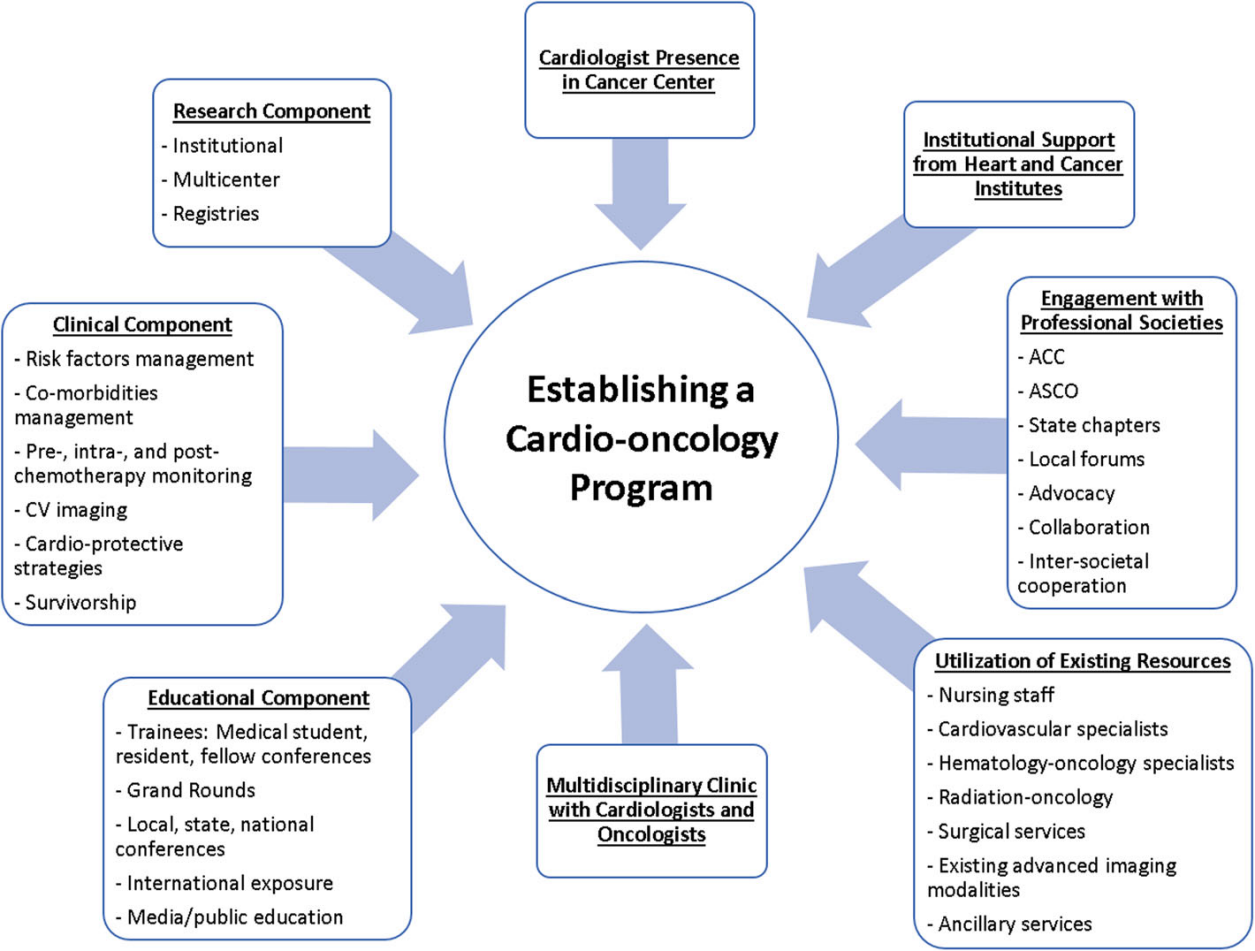
Central Illustration - “Key Components to Establishing a Successful CO Program”. This figure shows the integral components included in the build-out of this cost effective cardio-oncology program

We established four basic components as the foundation to develop the program (Fig. 1).

#### Clinical program

The project was presented to the leadership of both the Heart and Vascular Center and the Cancer Center, in order to address the following: associations between cancer and heart disease, the growing population of cancer survivors who exhibit CV disease as a major factor affecting morbidity and mortality [5, 11], growing evidence of CV effects of cancer-related therapies, and need for surveillance and co-management of CV disease before, during, and after cancer treatment [6]. Obtaining support from institutional leadership was critical to secure the basic resources needed to successfully run this program.

The goal was to optimize utilization of existing resources, in order to meet the unique CV surveillance needs of cancer patients: the authors agreed to utilize existing resources in nursing staff, CV specialists, hematology-oncology specialists, radiation oncology, surgical services, existing advanced imaging modalities, and ancillary services.

We began offering CO clinic services for one, half-day per a week. However, the increased patient need rapidly led to expanding those services to two, and finally three, full days of CO clinic per week (functioning via half of the day at the Cancer Center, and half of the day at the Heart and Vascular Center [12], each day offered). This schedule has allowed for interaction with the oncology staff, Infusion Center, Survivorship Clinic, and cardiovascular services. The authors utilized protocols from their own [13] and other institutions [14], as well as guidelines from ASE [15], ESC [16], and ASCO documents [17].

The authors focused efforts in the management of CV co-morbidities of cancer patients undergoing treatment, aggressive risk factors modification, and co-management of CV effects of cancer-related therapies (including effects of anthracyclines [18, 19]), HER2 targeted therapies [20–22], tyrosine kinase inhibitors (TKIs) [23], proteasome inhibitors [24–26], 5-FU [27], immune check point inhibitors (ICI) [28, 29]), effects of radiation therapies [30, 31], and utilization of imaging modalities in CO [13, 15, 32] as initial focal points.

#### Education program

The program included organized educational sessions with internal medicine, cardiology, and oncology services. This included: lectures for medical students, residents, and fellows with an introduction to the core clinical focuses of CO, “lunch and learn” sessions, Grand Rounds at the local and state levels, and educational symposia at the local and state levels.

Lectures by the authors were presented at international cardiology/imaging conferences in both Central and South America in order to promote and advance education and knowledge in the field of CO and increase the exposure for the program. The authors also engaged in public education regarding CO through participation and interviews with newspapers, radio, and television. This helped to amplify the presence of the program in the local market, as well as increase visibility with prospective referral sources.

#### Engagement with professional societies

A critical aspect of establishing a successful program in CO is to integrate the program members with existing professional societies. Participation in conferences, committees, and engaging with colleagues from different geographic areas, provided insight for multiple pathways critical to a successful program. The next step was to reproduce this integrated structure at the state and local level.

Active participation at the ACC Cardio-Oncology Advocacy Work Group was also a critical aspect of the program. The authors worked with the ACC- Florida Chapter to initiate and launch the Chapter Cardio Oncology Committee. This helped expand knowledge and education in CO, for the state of Florida. The authors also worked and integrated with the Florida Chapter of ASCO (FLASCO) in order to garner better understanding and cooperation between cardiologists and oncologists, and became active members of the International Cardio Oncology Society (ICOS).

#### Research component

In order to develop a comprehensive CO program, it is critical to make research a centralized focus. This allows for further clinical development of the program itself, and allows for its insertion within the CO community at-large. As such, the following actions were taken:

o Development of data collection for clinic patients.

o Cooperation with oncologists in existing protocols.

o Joining SURVIVE Registry (Washington University Center of Cardio Oncology) for cooperation and development of databases in CO and to have a platform for clinical trials and started onboarding for UPBEAT clinical trial.

o Started and completed cooperation projects with Florida Chapter of ACC and FLASCO for cooperation in contributing to filling the gaps at state level. Results of research projects have been communicated and published elsewhere.

A prospective data collection and a retrospective chart review was also conducted to determine the demographics of the patients seen in this clinic, the reason for referral, exposure to cardio-toxic agents, cardio-protective strategies implemented, and the overall outcomes of patients with cardiac dysfunction or those who presented at-risk for cardiac dysfunction. Univariate analysis was performed on the data obtained from the CO clinic patient population, and is reported via mean or frequency information, as statistically appropriate, per metric.

## Results

### Demographics and most common cancer types

A total of 474 patients were seen during the first 25 months of operation at the CO clinic of Cleveland Clinic Florida. This resulted in a combined 1422 patient visits. Mean age was 64.1 (range 26–96). Among this patient group: 171 (36%) were male, 303 (64%) were female. Referrals to the CO clinic were predominately from the hematology-oncology service (*n* = 379, 80%), followed by internal medicine (*n* = 80, 17%), and other (*n* = 15, 3%).

**Fig. 2:**
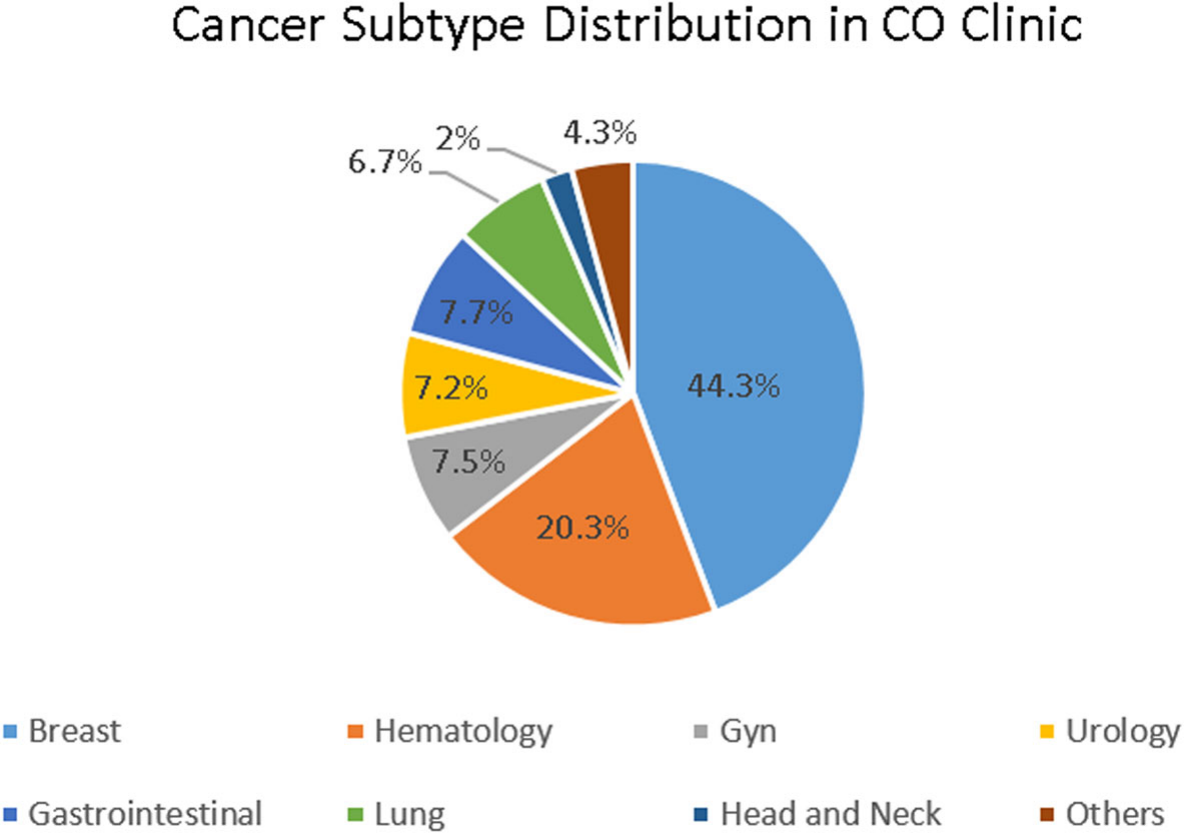
“Cancer Subtype Distribution in CO Clinic”. This chart shows the relative proportion of cancer diagnoses among the patient population seen in the cardio-oncology clinic, over the course of 25 months. The most common diagnosis was breast cancer (45%)

The most common underlying cancer diagnosis was breast cancer (*n* = 214, 45%), followed by hematological malignancies (mostly multiple myeloma, leukemia, and lymphoma) (*n* = 104, 22%), gastrointestinal/colorectal malignancies (*n* = 44, 9.3%), genitourinary (*n* = 35, 7.4%), lung (*n* = 34, 7.2%) and gynecological malignancies (*n* = 34, 7.2%) **(**Fig. 2**).**

### Referrals: when and why were patients referred

In Table 1, a summary is presented, describing the timeframe in which patients were referred to the CO clinic, relative to their cancer treatment (note: 12 patients descriptively qualified for two categories and were tabulated as such).

**Table 1:** Therapeutic Modalities Received by CO Clinic Patients

Patient CO Visit with Respect to Cancer Treatment	Number of Patients
Prior to 1st course of chemo	66
During 1st course of chemo (including year of trastuzumab)	85
1st course of chemo completed – seen prior or during 2nd course of chemo	71
Chemo completed/discontinued – currently treated with endocrine therapy or immunotherapy	23
Never received chemo – currently treated with endocrine therapy or immunotherapy	28
Chemo completed/discontinued – not being actively treated for cancer	156
Never received chemo – history of chest radiation therapy	29
N/A, surgery only, or patient unsure	31
Total Patients^a^	474

This chart shows the frequency of therapies received by the patient population seen in the cardio-oncology clinic, over the course of 25 months. ^a^Of these, 12 patients received > 1 therapy type

The reason for referral to the CO clinic was highly varied, and can be stratified into four broad categories: 1) pre-existing cardiovascular conditions; 2) presentation of new symptoms; 3) referral due to *combined* pre-existing cardiovascular conditions and new symptoms, and 4) asymptomatic referral (for risk stratification, pre-operative evaluation, etc.). The most common, new cardiac symptoms included: shortness of breath/dyspnea on exertion (*n* = 143), chest pain (*n* = 93), palpitations (*n* = 84), edema (*n* = 27), syncope/pre-syncope/dizziness/hypotension (*n* = 22).

The most common cardiovascular co-morbidities among this patient group included: hypertension (*n* = 269, 56.7%), dyslipidemia (*n* = 237, 50%), diabetes (*n* = 67, 15%), atrial fibrillation (*n* = 48, 10%), tachycardia/pacemaker (*n* = 85, 18%), coronary artery disease (*n* = 44, 9.3%), heart failure (HF) (*n* = 50, 10.5%), deep venous thrombosis/pulmonary embolism (DVT/PE) (*n* = 31, 6.5%) **(**Table 2**).**

**Table 2:** Cardiovascular Co-Morbidities Among CO Patients

Co-Morbidity	n (%)
Hypertension	269 (56.7)
Dyslipidemia	237 (50)
Diabetes	67 (14)
Tachycardia/pacemaker	85 (10.1)
Atrial fibrillation	48 (18)
Syncope	16 (3.4)
CAD	44 (9.3)
CHF	50 (10.5)
VTE/DVT/PE	31 (6.5)
Total Patients^a^	474

This table shows the frequency and proportion of cardiovascular comorbidities among the patients seen at the CO clinic. ^a^Patients presented with multiple comorbidities. (*CAD* Coronary artery disease. *CHF* Congestive heart failure. *VTE* Venous thromboembolism. *DVT* Deep venous thrombosis. *PE* Pulmonary embolism)

### Most common cancer therapies in our clinic

-Most commonly included radiation in 192 patients (40%), doxorubicin in 127 patients (26.8%), cyclophosphamide in 94 patients (20%), trastuzumab in 85 patients (18%), proteasome inhibitors in 29 patients (6.1%), TKI in 48 patients (10.1%), cisplatin in 37 patients (7.8%), rituximab in 30 patients (6.3%), 5 FU/capecitabine in 41 patients (8.6%), androgen deprivation therapy in 11 patients (2.3%), and ICI in 32 patients (6.7%).

- Patients in receipt of chest radiation were mostly breast cancer and lymphoma patients.

- Radiation was included in this category although it was an infrequent cause for the referral.

### Cardiovascular complications and impact inpatient management

-One hundred and fifty six patients were referred for evaluation during the course of their chemotherapy or treatment.

- Twenty two patients had documented temporary interruption in their cancer treatment for potential cardio-toxicity risk until CO evaluation was completed. In these instances, the patients were permitted to continue chemotherapy after initial CO evaluation and treatment was completed.

- Thirty four patients had evidence of subclinical left ventricular (LV) dysfunction by global longitudinal strain decrease (normal value: < 18%), abnormal biomarkers (normal value: pro BNP > 300 pg/ml or troponin > 0.04 pg/ml), or mild reduction of LVEF < 10% by 2-D echo.

These patients had received treatment with doxorubicin, trastuzumab, TKIs, and proteasome inhibitors and were treated with cardio-protective medications, including beta blockers (mostly carvedilol) and angiotensin converting enzyme inhibitors (ACE-) or angiotensin receptor blockers (ARB). Out of the 34 patients, 22 had complete and 12 had partial improvement of their baseline abnormality, had stable clinical condition and were subsequently able to continue treatment.

- Sixteen additional patients had their chemotherapy modified or discontinued due to cancer therapy related cardiac dysfunction, defined as a drop of LVEF > 10% to < 53%, confirmed by repeat echocardiogram 3–4 weeks later.

- Fourteen patients, were evaluated for suspected ICI myocarditis secondary to immunotherapy. Of those with suspected myocarditis, 7 had the diagnosis ruled out by negative biomarkers, and negative CMR or endo-myocardial biopsy and were permitted to continue treatment; 5 patients required full discontinuation of ICI due to therapy-related confirmed myocarditis: two by clinical presentation and confirmed with endo-myocardial biopsy, and 3 by clinical presentation, positive biomarkers, and confirmation by CMR with typical myocarditis findings. Two additional patients presented with delayed onset cardiomyopathy, CHF after prolonged ICI treatment and also discontinued their ICI treatment.

- Eighty two patients had part of their treatment regimen modified or discontinued due to non-cardiac related toxicity, including: infection, disease progression, renal toxicity, cytopenias, neuropathy, allergic reaction, and pneumonitis.

-There were 30 deaths in our study population: 26 from documented progression of cancer, 4 with undocumented cause, presumably due cancer. There were no documented cardiac deaths in our cardio oncology population during the 25 months of data collection.

### Utilization of cardiovascular testing

In order to ascertain the extent of cardiovascular health, the following testing modalities were utilized as part of the CO clinic services:

− 2-D echocardiogram: 472 patients (99.5%), biomarker panels (troponin and pro BNP): 254 patients (53.5%), 3-D echocardiogram and global strain (GLS): 142 patients (33%), stress test/nuclear/stress echo: 148 patients (31%), Holter/event monitor: 83 patients (17.5%), cardiac catheterization: 21 patients (4.4%), and CMR: 22 patients (4.6%) **(**Table 3**).** The high utilization rate of cardiovascular testing in our cardio oncology clinic reflects the high cardio vascular risk and pre-existing cardiac co-morbidities in these cancer patients.

**Table 3:** Cardiovascular Testing Performed CO Patients

Testing Modality	n (%)
Echocardiography	472 (99.5)
Biomarkers	254 (53.5)
Stress testing	148 (31)
3-D and strain imaging	142 (30)
Holter/Event monitor	83 (17.5)
Cardiac catheterization	21 (4.4)
Cardiac MRI	22 (4.6)
Total Patients^a^	474

This table shows the frequency and proportion of cardiovascular testing modalities used among the patients seen at the CO clinic. ^a^Multiple testing modalities may have been used, per patient

All patients received 2-D imaging – 3-D and strain imaging was utilized in most cases of HER2 therapies and anthracyclines. The lack of consistency in use of 3-D and strain imaging until recently was secondary to lack of insurance coverage and denials for many patients.

- The most common echocardiographic abnormality was a stage I diastolic abnormality (*n* = 123); 24 patients presented with stage II-III diastolic abnormality. Twenty three patients exhibited RSVP > 45 mmHg, consistent with elevated right sided pressures; 24 patients had, at least, moderate valve heart disease.

- Biomarkers (pro BNP and troponins) were measured at baseline in patients treated with anthracyclines, HER2 therapies, proteasome inhibitors, ICIs, and in other selected patients, if clinical presentation warranted it. Serial follow-up biomarkers were utilized when there were new onset cardiovascular symptoms, initial abnormal biomarker results, or abnormal results in non-invasive imaging tests.

- Of the patients seen in the CO clinic, 41 exhibited B-type natriuretic peptide (BNP) levels > 300 pg/mL; only 15 exhibited abnormal troponin levels.

## Discussion

### Rationale for a program compared to previous models

Resources available to implement a CO program can be limited. Commitment to team work with dedicated existing clinical resources, education, advocacy, and research are the main components that were integral to the observed success in starting and sustaining a CO program.

We described the primary rationale for starting a program, the key players for institutional support, the four key components to develop a sustainable program, and the patient population seen in our CO clinic, with significant cardiovascular co-morbidities and the associated high utilization of common diagnostic cardiovascular modalities in these patients.

Sulpher, et al. [33] reported their experience in a multidisciplinary CO clinic (2008–2013), utilizing a model that differed significantly from ours. Of their patient group, 43% exhibited reduced LVEF, HF, or cardiomyopathy at time of referral, and 51% of patients had at least one episode of decreased LVEF between 10 and 20% from baseline. These metrics point towards a patient population with advanced cardiovascular disease, referred to a CO clinic at a much later stage of disease, likely reflecting a pattern different from the more contemporary practice of early referral and preventive care prior to clinical deterioration.

In another study by Barros-Gomes, et al., [34] the authors described the Mayo Clinic experience, wherein a comprehensive CO program was established with multiple providers and physician extenders. Our program has started with a much smaller infrastructure, and aims to be a model that can be used in other centers with more limited resources.

Most recently, Sundlöf, et al. [35] reported on developing a CO program in a community hospital setting, which represents a growing trend in the United States. Our program includes elements of both community-based and academic-based programs, with the strengths of each setting, and with a strong emphasis in advocacy, education, inter-institutional networking, and building a CO community to improve access to care. Our model succeeded in part due to our strong integration to the existing state and national cardio oncology community.

Our CO clinic population had a high prevalence of cardiovascular comorbidities, with a high incidence of risk factors and pre-existing CV disease, consistent with a high-risk population as per ASCO document on CV risk stratification [11], and represents a population that will likely benefit from early screening and cardio-protective strategies.

There is a potential for early intervention strategies to minimize CV effects of cancer-related therapies, although clinical trial evidence is still scarce. Results of small size randomized trials including PRADA [36], MANTICORE [37], the CECCI trial [38] and a larger study by Guglin et al [39] have shown modest but statistically significant benefit on LV function for cardio protective strategies with selected beta blockers and ACE-, particularly in high risk populations [38, 39].

Our CO population’s high prevalence of risk factors factors resulted in a high utilization rate of CV testing. We are not aware of cardiovascular testing utilization rates as reported in previous papers on CO programs. This may further indicate a potential financial benefit inherent to CO programs: the downstream testing generated by CO should allow administrators to justify and support these programs. Our CO program had significant utilization of non-invasive multi-modality imaging and referred for invasive testing like cardiac catheterization and electrophysiology testing/procedures in selected patients with either CV co morbidities or complications from their cancer treatment. The wide utilization of CV testing in this population emphasizes the need for a team approach to rapidly identify the CV requirements of these patients and to minimize disruption of their cancer treatment.

The CO program had an immediate impact in patient care: it allowed a number of patients to complete their cancer treatment when otherwise that treatment was being held due to CV concerns. Additionally, it allowed for modification of cancer treatment when needed, and provided cardiovascular advice, care, and treatment to patients who needed to have their treatment delayed or interrupted secondary to cardio-toxicity of cancer related therapies. Similarly, it provided support and treatment of cardiovascular risk factors and cardiovascular co-morbidities during cancer treatment, and provided cardio protective strategies that resulted in satisfactory completion of planned cancer treatment.

We defined the success of our program by the ability to start and maintain a new service line in our institution, meeting the needs of a growing patient population, our strong cooperation with the oncology department, the achieved goal of bringing awareness of cardiovascular health in cancer patients, and by specific clinical benefits to multiple patients as described herein.

The ability to maintain a Cardio Oncology service line is multifactorial and challenging. It needs dedicated commitment from the Cardiology and Oncology departments, financial support from administration, financial self-sustainability and new sources for growth. New technologies like telemedicine, now with wide spread use since the onset of the COVID 19 pandemic, will likely become a permanent alternative to in-person office visits. Employment of telemedicine may improve access to specialty care and expand the reach of CO programs. Virtual video/telephone visits may play a critical role in the ability to monitor short and long-term CV complications of cancer treatment [40, 41].

### Our cardio oncology structure

Our institution is a tertiary center with a full range of cardiovascular services, including an Amyloidosis Center and an active Heart Failure and Transplant Center. There was, however, no existing CO program and there were no financial resources allocated for a CO program. Most of our growth came from oncology referrals, and active involvement with existing CO sections/councils at the ACC, and International Cardio Oncology Society (ICOS). Therefore, this model can be reproduced at both academic and community practice settings.

The associated cancer center is a tertiary cancer center with offering services with clinical oncologists, radiation oncologists, and surgeons. The breast oncology and hematology oncology services accounted for 67% of the CO referrals. A bone marrow transplant and CAR-T cell program is scheduled to open later in 2020.

The survivorship clinic is operated by oncology advanced registered nurse practitioners (ARNPs), who refer the patients to CO for cardiovascular assessment and follow-up. The breast oncology survivorship clinic is the leading survivorship clinic at our cancer center.

Until now, only one cardio-oncologist has seen all patients in conjunction and consultation with multiple oncologists. We have the support of cardiac imaging, electrophysiology, and heart failure specialists that collaborate in the management of many of these patients. We have recently incorporated a cardiology fellow, on a once per week rotation in CO, and two heart failure faculty members, who will collaborate and will become part of the CO clinic. We will have a dedicated CO research fellow, and will recruit an additional cardio oncology faculty member. We have educated our nurses, administrators, and schedulers on the needs of our CO clinic, facilitating adding urgent slots, and have established longer CO office visit times to allow detailed review of all oncology records during each CO encounter.

Our active engagement with the cardio oncology community started with the Global Cardio Oncology Summit (GCOS) meeting in 2015, and followed since with all GCOS, Memorial Sloan Kettering Cancer Center annual CO conferences, and ACC meetings. We have also become active participants in CO with the ACC and ICOS, and collaborated in starting the ACC Florida Chapter Cardio Oncology Committee, active participants with the Florida ASCO chapter, and with the ACC Cardio Oncology Council Advocacy Work Group. We also gave CO presentations and talks at many cardiology conferences and congresses in Central and South American countries. Active involvement with professional societies and their CO sections/councils, is the best way for a new program to participate, learn from leading experts, keep pace with recent advances, and to grow a program and network.

### Plan for continuous growth

It is our expectation that the program will continue to grow in the four basic components:

#### Clinical

1) We will complete protocols with guidelines for specific referral scenarios for oncologists, under the conditions where the CO clinic may have the most impact in co-management. We will subsequently evaluate for differences in referrals based on the new standardized protocols, 2) A CO in-patient consultation service will be established, and 3) With the expected launch of the bone marrow transplant and CAR-T cell program, we will add time and staff to meet these demands.

#### Education

1) We have started a CO rotation for cardiovascular fellows, wherein they will rotate through the CO clinic, 1 day per week, during their clinic rotation, 2) We will establish a basic curriculum in CO for all fellows to complete during their cardiovascular disease fellowship, and 3) Two faculty from the heart failure service will integrate to the CO program, on part-time basis, with plans to target additional CO faculty recruitment.

#### Engagement with professional societies

1) Continue to advance with multiple projects with Florida Chapter ACC, Florida Chapter of ASCO, the ACC Cardio Oncology Council and ICOS.

#### Research

Current involvement in manuscripts, abstracts, case reports, and participation in a national Registry (SURVIVE), onboarding process for UPBEAT clinical trial. We plan on 1) implementing at least two clinical trials for 2020, one multi institutional, one institutional based, and 2) to develop new institutional databases for prospective research projects. We currently have active participation in research projects in advocacy, education, and access to care in CO. Started and completed new projects with new Cardio Oncology Multi State and International ACC, ASCO, ICOS Collaborative Network.

### Reproducibility of this model for starting new programs

We aim for our CO program model to be reproduced by other institutions with the focus on the four basic components listed herein. Each new program will need a cardiologist and an oncologist within the institution who can champion and lead the initiative. We aim to expand our program to regional sister institutions, and we will continue to work hard and collaborate with the Florida Chapter of the ACC and ASCO and with the ACC CO Council Advocacy Work Group to improve education, awareness and clinical access to cardio oncology services.

We were able to start and sustain the program with the use of existing resources: involvement by the leadership of both the CV and oncology departments was critical to efficiently utilize administrative, clinical, and existing ancillary staff resources in order to run the CO program. As such, this clinic became fully operational without the need of large budget assignments or additional investment. The Heart and Vascular Institute support was displayed by allowing the cardiologist to spend an increasing amount of the time in the CO clinic, blocking dedicated time for CO, allowing longer appointment times for CO patients, and assigning cardiology nurses to the cancer center for the CO clinic. No additional salaries or hiring occurred when the clinic was started. The cancer center allowed the use its facilities, twice per week, for the CO clinic operations, and provided support for research with existing cancer center research staff. The implementation of cardiology services provided at the cancer center was instrumental for rapidly developing interdisciplinary cooperation and subsequent growth of the program.

In our study population, over 80% of referrals came from the hematology-oncology service, further highlighting the critical importance of the oncologists’ full involvement in a successful program. It is also critical for the CV specialist to have a physical presence at the cancer center. This presence was observed to stimulate cooperation and discussion of complex cases. Additional resources included marketing with local and regional media (radio, television, newspapers) which provided additional exposure and referrals.

The financial impact of the CO clinic can be extrapolated from the revenue generated by an additional 474 new patients (1422 total visits) and the large number of cardiovascular testing described in Table 3, over 2 years. Additional downstream testing is not reflected in these projections. This CO program was financially sustainable given the revenue generated for the institution, while also getting a new service line that improved patient care.

Finally, there is no better way to grow, learn, develop protocols, and have an impact in the community than having an active participation in existing professional societies. The ACC and ASCO state Chapters, the Cardio Oncology Section of the ACC and ICOS provide a very rich platform to achieve these goals. It is equally important to develop educational activities at the hospital and community level to assure the program’s presence is known and that the cardio-oncologist becomes a resource available to all cardiology and oncology colleagues for the cardiovascular care of cancer patients.

The complex clinical presentations, as well as the volume of patients referred to the CO clinic within the first 25 months of operation, indicates that there is a critical need for these services.

## Conclusions

- This model shows how to establish a de-novo cardio-oncology program with limited resources.

- Four components were implemented as the foundation to the program: clinical program, education, professional society engagement, and research.

- Active involvement with professional societies, from early stages, facilitates growth, education, and provides a platform for collaboration.

- We propose that this model can be reproduced in a variety of different practice settings and by incorporating more educated cardio oncology providers to the healthcare workforce may improve access to care.

## Data Availability

All data generated or analyzed during this study are included in this published article.

## Abbreviations

CO: Cardio-oncology
CVD: Cardiovascular diseases
CV: Cardiovascular
CMR: Cardiac magnetic resonance
LVEF: Left ventricular ejection fraction
ACC: American college of cardiology
ASCO: American society of clinical oncology
FLASCO: Florida chapter of ASCO
ICOS: International cardio oncology society
ACE: Angiotensin converting enzyme inhibitors (ACE-)
ARB: Angiotensin receptor blockers.
HF: Heart failure
DVT/PE: Deep venous thrombosis/pulmonary embolism
ICI: Immune check point inhibitors
ARNP: Advanced registered nurse practitioners
